# Multinomial logistic regression model to assess the levels in trans, trans-muconic acid and inferential-risk age group among benzene-exposed group

**DOI:** 10.4103/0019-5278.72238

**Published:** 2010-08

**Authors:** A. Mala, B. Ravichandran, S. Raghavan, H. R. Rajmohan

**Affiliations:** Regional Occupational Health Centre (Southern), Indian Council of Medical Research, Bangalore, India

**Keywords:** Benzene, multinomial logistic regression, petrol filler, t, t-MA

## Abstract

There are only a few studies performed on multinomial logistic regression on the benzene-exposed occupational group. A study was carried out to assess the relationship between the benzene concentration and trans-trans-muconic acid (t,t-MA), biomarkers in urine samples from petrol filling workers. A total of 117 workers involved in this occupation were selected for this current study. Generally, logistic regression analysis (LR) is a common statistical technique that could be used to predict the likelihood of categorical or binary or dichotomous outcome variables. The multinomial logistic regression equations were used to predict the relationship between benzene concentration and t,t-MA. The results showed a significant correlation between benzene and t,t-MA among the petrol fillers. Prediction equations were estimated by adopting the physical characteristic viz., age, experience in years and job categories of petrol filling station workers. Interestingly, there was no significant difference observed among experience in years. Petrol fillers and cashiers having a higher occupational risk were in the age group of ≤24 and between 25 and 34 years. Among the petrol fillers, the t,t-MA levels with exceeding ACGIH TWA-TLV level was showing to be more significant. This study demonstrated that multinomial logistic regression is an effective model for profiling the greatest risk of the benzene-exposed group caused by different explanatory variables.

## INTRODUCTION

### Study Design and Data Sources

Benzene is a constituent of motor fuel, a byproduct of combustion, automobile exhaust and cigarette smoke, which is a widespread environmental pollutant. Because it is classified as a human carcinogen, monitoring of benzene in the environment and its biomarkers is of importance. Trans-trans-muconic acid (t,t-MA) is one of the biomarkers for benzene exposure. In this study, t,t-MA was estimated in the urine samples of petrol filling station workers from various petrol filling stations located in Bangalore. It is important to study the health risk factors among petrol filling station workers viz., manager, cashier and petrol filler. Irrespective of their designation, all the three were involved in that occupation. There is a rapid increase in usage of vehicles, which leads to a greater benzene exposure among this group. Classification and prediction are the more common practices in applied medical research. Mathematical model is widely used for prediction of exposed outcomes. Discriminate analysis is mainly used for classification and logistic regression and the dependent variables in binary or strict, with two categories. In a few studies, the relative predictivity of these methods was employed as an outcome variable that had more than two groups with unequal sizes. These models have been investigated when reducing bias by promoting the efficiency of the parameter estimation when the dependent variable has more than two groups. In this study, the multinomial logistic regression model was employed to identify the benzene-exposed group at the greatest risk of higher levels of t,t-MA levels, age groups and experience in years. The proposed objective of the study was to determine the likelihood of workers to have exceeded the t,t-MA values.

## MATERIALS AND METHODS

A total of 117 urine samples were collected from petrol filling station workers in Bangalore city, covering the residential, commercial and industrial areas. Urine samples were sent to the laboratory and were analyzed for t,t-MA using a high-performance liquid chromatography-ultraviolet system. The standard improved method followed by NIOH has been followed for the estimation of t,t-MA. Standard methods followed by Ducas *et al*. in 1990 and 1992 have been referred for the study.[1]

### Data Management

The entered data were randomly checked and matched to derive consistency and validity. The final analysis was performed using SPSS, version 10. In this study, the petrol filler workers explored for t,t-MA values were classified as exceeding ACGIH TWA-TLV limits and below the same, recoded as 1 and 2, respectively. Also, the age groups and experience in years changing pattern was determined by parametric estimation through odds ratio (OR). The age groups were classified into three categories viz., up to 24 years, above 25–34 years and more than 35 years, and were coded as 1–3, respectively.

The experience in years was classified into four categories viz., up to 5 years, above 6–10 years, above 11–15 years and more than 16 years, and were coded as 1–4, respectively.

The relationship between the risk-dependent variable and each of the three explanatory variables are shown in Table 1.

**Table 1:** Distribution of benzene-exposed workers according to t,t-MA values, age group and experience in years

Job category	t,t-MA				Age group						Experience in years							
	Below ACGIH TWA-TLV level		Exceeding ACGIH TWA-TLV level		≤24		25–34		≥35		≤5		6–10		11–15		≥16	
	No.	%	No.	%	No.	%	No.	%	No.	%	No.	%	No.	%	No.	%	No.	%
Cashier (*n* = 41)	31	75.6	10	24.4	7	17.1	24	58.5	10	24.4	16	39.0	15	36.6	5	12.2	5	12.2
Petrol filler (*n* = 64)	34	53.1	30	46.9	25	39.1	23	35.9	16	25.0	36	56.3	15	23.4	9	14.1	4	6.3
Manager (*n* = 12)	11	91.7	1	8.3	1	8.3	1	8.3	10	83.3	6	50.0	3	25.0	-	-	3	25.0

To describe categorical-dependent variables and one or more categorical or dichotomous or continuous explanatory variables, logistic regression was found suitable if dependent is strict with two categories.

The objective of this study was to employ multinomial logistic regression (MLR), which may be more efficient and reliable to obtain the probability estimation of the concerned exposed group. In addition, MLR explores estimation of the net effects of a set of explanatory variables on the dependent variable.[2–4]

### Data Analysis

The MLR model involves categorical-dependent variable (more than two) Y, e.g. three categories of exposed group and three explanatory variables x_1_, x_2_and x_3_(x_1_ = t,t-MA, x_2_ = age groups and x_3_ = experience in years).

Let P_1_ = probability of cashier at risk (Y = 1), P_2_ = probability of petrol filler at risk (Y = 2), P_3_ = probability of manager at risk (Y = 3). The modality of MLR relates to the log odds (or logit) of Y to the explanatory variable x_i_ in linear form as:

Pi = A + Pxi

(1) $$\mathrm{Probit}\left(\mathrm{Pi}\right)\;=\;\mathrm{intercept}\;+\;R.\;\mathrm{co-effi}\left(\mathrm{xi}\right)$$

The model explores

(2) $$\mathrm{Prob}\left(y=\;j\right)\;=\;\frac{\mathrm{e\sum \beta jkxk}}{1\;+\;\mathrm{\sum e\sum \beta jkxk}}$$

(3) $$\mathrm{Pij}\;=\;\log\frac{P(\mathrm{∆i})}{P(\mathrm{∆3})}$$

i,j,k >0 i = exposed group 1–3, j = t,t-MA, age group, experience and k = age groups 1–3, experience 1–4 and t,t-MA 1–2

- The analysis was made to identify the risky exposed benzene group. Overall, explanatory variable age group below ≤ 24 years (*P* < 0.01, OR = 40.7, 95% confidence interval [CI] 2.46, 673.4) was highly significant with the cashier group. Also, age group between 25 and 34 years (*P* < 0.001, OR = 72.9, 95% CI 5.69, 933.74) was highly significant with the cashier group.
- The variable t,t-MA values exceeding the ACGIH TWA-TLV (*P* < 0.01, OR = 24.51, 95% CI 2.346, 673.4) was highly significant with the benzene-exposed group of petrol filler. Also, age group 24 and between 25 and 34 years (*P* < 0.01, OR = 64.31, 95% CI 4.408, 938.214; *P* < 0.01, OR = 39.51, 95% CI 3.20, 487.677) were highly significant with the petrol filler group, respectively [Table 2].

**Table 2:** Multinomial logistic regression models exploring significant petrol filler workers with respect to their explanatory variables

Job category	Cashier				
Age group	No.	%	*P*-value	OR	95% CI
≤24	7	17.1	0.010	40.7	2.46, 673.4
25–34	24	58.5	0.001	72.9	5.69, 933.74

**Job category t,t-MA Age group**	**Petrol filler**				
	**No.**	**%**	***P*-value**	**OR**	**95% CI**

Exceeding ACGIH TWA-TLV	30	46.9	0.008	24.51	2.346, 256.06
≤24	25	39.1	0.002	64.31	4.408, 938.214
25–34	23	35.9	0.004	39.51	3.20, 487.677

As per the above equation (3), the result shows

Predicted logit (Y cashier)

= -0.474 + 3.707

(age group ≤ 24 years)

Predicted logit (Y cashier)

= -0.474 + 4.289

(age group 25-34 years)

Predicted logit (Y petrol filler)

= -1.235 + 3.199

(t,t-MA >500 mg/g)

Predicted logit (Y petrol filler)

= -1.235 + 4.164

(age group ≤ 24 years)

Predicted logit (Y petrol filler)

= -1.235 + 3.676

(age group 25-34 years)

- Overall, the chi-square test of proportional odds assumption was significant (*P* < 0.001). All other variables show no significant results.

## CONCLUSION

- Generally, logistic regression analysis (LR) is a common statistical technique, which could be used to predict the likelihood of categorical or binary or dichotomous outcome variables.
- Reference group was fixed based on the number of cases. In this study, the manager group was treated as the reference group.
- The MLR supported the statistical significance of the two independent variables in two exposed groups: cashier and petrol filler for different age groups. In the categorical outcomes, LR is more flexible and less restrictive than discriminant function analysis and log-linear models.[5]
- Interestingly, there was no significant difference among experience in years, petrol filler and cashier having more occupational risk in the age group of ≤ 24 and between 25 and 34 years. Among petrol fillers, the t,t-MA levels with exceeding ACGIH TWA-TLV level was showing higher significance. We found that MLR is an effective model for profiling greatest risk of the benzene-exposed group caused by different explanatory variables.
